# Predicting breast cancer risk and its association to biopsychosocial factors among Taiwanese women with a family history of breast cancer: an investigation based on the Gail model

**DOI:** 10.1186/s12920-025-02149-w

**Published:** 2025-05-16

**Authors:** Sabiah Khairi, Nur Aini, Lalu Muhammad Harmain Siswanto, Min-Huey Chung

**Affiliations:** 1 School of Nursing, College of Nursing, Taipei Medical University, Taipei, Taiwan; 2 Department of Nursing, Faculty of Health Sciences, Universitas Muhammadiyah Malang, Malang, Indonesia; 3 Mataram Health Training Center, Indonesian Ministry of Health, Mataram, Indonesia; 4 Department of Nursing, Shuang Ho Hospital, Taipei Medical University, New Taipei City, Taiwan

**Keywords:** Gail model, Biological factors, Psychological factors, Social factors, Risk assessment, Breast cancer

## Abstract

**Background:**

First-degree relatives with breast cancer have a two-fold higher risk than women without a family history. The Gail model approach has been employed in numerous studies to investigate the risk of breast cancer among women in a variety of countries. Nevertheless, the studies investigating the correlation between the level of breast cancer risk and biopsychosocial factors among Taiwanese women with a family history of breast cancer (FHBC) are limited. By using the Gail model, we explored the breast cancer risk score and its relationship to biopsychosocial factors among Taiwanese women with FHBC.

**Methods:**

The present study was a cross-sectional study from secondary data of the Taiwan Biobank from 2008 to 2018. Self-reports were conducted to determine biopsychosocial factors. A total of 3,060 women aged 35–70 years with and without FHBC were considered eligible for enrollment. The Gail model, which utilizes six questions, was used to estimate individual five-year absolute breast cancer risk. Women with scores at least 1.66% and above were categorized as high risk. In addition, we performed bivariate and multivariate logistic regression analysis using SPSS version 27 to predict the associations between biopsychosocial factors and the risk of breast cancer based on the Gail model. All analyses were stratified by age.

**Results:**

Among the 3,060 Taiwanese women, there was a statistically significant difference in breast cancer risk score between the groups with and without FHBC (*p* = < 0.001), stratified by age, of which 574 in FHBC group (34.2%) were identified as having a high breast cancer risk based on the Gail model. Furthermore, six out of 15 biopsychosocial factors were significantly associated with breast cancer risk in women under 50 years of age, while seven factors showed significant associations in women aged 50 years and older. Logistic regression analysis identified five biopsychosocial factors as consistent and significant predictors of breast cancer risk in women aged 50 years and older, highlighting this group as particularly vulnerable.

**Conclusions:**

This study concludes that the Gail model identifies Taiwanese women who have a higher estimated risk of breast cancer based on cross-sectional data. Various biopsychosocial factors are associated with higher risk estimates in this population particularly in older women. Professionals can assist women in recognizing risk factors beyond the inevitable risk by encouraging regular screenings, positive behavior, and health promotion.

## Background

Breast cancer remains one of the most prevalent cancers worldwide, with first-degree relatives having a two-fold higher risk than those without a family history. According to the World Health Organization (WHO), 2.3 million (11.7% of total cases) women were diagnosed with breast cancer in 2020, and 685,000 (6.9%) deaths globally [1]. In 2022, The global cancer burden using the GLOBOCAN reported lung cancer was the most commonly detected cancer, accounting for about 2.5 million new cases, which is equivalent to one out of every eight cancers worldwide (12.4% of all global cancers). It was followed by breast cancer (11.6%), colorectal cancer (9.6%), prostate cancer (7.3%), and stomach cancer (4.9%). Among women, breast cancer was the most prevalent form of cancer, accounting for both the highest number of cases and deaths. It was responsible for around 6.9% of all breast cancer-related fatalities [2]. In Taiwan, the Top 10 cancer incidence rate per 100,000 for carcinoma in situ and invasive cancers in 2021 was female breast with a total of 18,182 cases. resulting in approximately 2,913 fatalities [3]. In addition, breast cancer is frequently found among younger Taiwanese women aged 40–65, with stages 0 to II being the most prevalent at diagnosis [4, 5].

It is important to note that, each individual is a combination of biological, psychological, and social components that are more than the sum of their parts. Biopsychosocial factors which influence internal micro-environmental cues also contribute to the development of these alterations that are responsible for tumorigenesis [6]. Several biopsychosocial factors are associated with the development of breast cancer, including sociodemographic [7, 8], reproductive characteristics [9], lifestyle [10, 11], psychological stress, coping ability, and family history of breast cancer (FHBC) [7, 8]. According to age-standardized incidence and mortality data from the United Kingdom, where the incidence is highest in the world, women aged 50 suffer from the disease at a rate of approximately two per 1,000 per year, and it is the leading cause of death for women in the 40–50 age bracket [12]. On the other hand, breast cancer is uncommon in young women, occurring in only 4–6% of those under 40. However, it is still the most frequently diagnosed malignancy in this age group. In recent years, there has been a noticeable rise in BC cases among pre-menopausal individuals [13]. Young women are more likely to develop tumors that exhibit a higher prevalence of unfavorable clinicopathologic characteristics, such as higher histological grade, increased lymph node involvement, lower estrogen receptor (ER) positivity, and elevated Her2/neu overexpression. Additionally, they are often diagnosed at more advanced stages of the disease [13].

In relation to the family history, this factor is a well-established biological risk factor for breast cancer that contributes significantly to the risk of the disease (odds ratio: 1.71, 95% CI: 1.59–1.84) [9]. A different investigation, employing a substantial group of patients, revealed that women with two or more relatives who have previously had breast cancer have a 2.5-fold higher risk (95% CI: 1.83–3.47) of developing the disease [14]. These findings collectively indicate that older age and a family history of breast cancer as a crucial factor in breast cancer prevention screening [12, 15].

As previously pointed out, breast cancer is a malignant disease that primarily affects women and can be effectively prevented with early detection strategies. Early detection techniques can include screening tests, and breast self-examination to identify life-threatening diseases at their initial stages [16]. Over the last thirty years, numerous statistical models have emerged to evaluate breast cancer risk in both individuals and populations [17]. The Gail model discovered by Gail et al. [18] stands out as the most widely used for overall risk evaluation, capable of assessing a range of potential risk factors for both five-year and lifetime invasive breast cancer risk [18, 19]. Present models have the capability to provide a reasonably precise prediction of the likelihood of developing breast cancer, and they can adequately advise women if they possess an elevated risk over their lifetime [20]. The National Cancer Institute has created the Breast Cancer Risk Assessment Tool (BCRAT), an online calculator that has been validated to predict the risk of invasive breast cancer for women in the United States. This tool is based on the Gail Model, which has been validated in large samples specifically for white women [16]. The inclusion of family history is a crucial component of this instrument, as it is in most other breast cancer screening techniques. Women who receive a positive screening result might benefit from being referred to a specialized breast center for genetic counseling, additional testing, and treatment [16].

Numerous studies have investigated breast cancer risk based on the Gail model approach in women of reproductive age in Nigeria [21], among secondary school teachers in Saudi women [17], patients diagnosed with non-proliferative lesions who have had a breast biopsy in Iraq [22], and patients with invasive breast carcinoma in USA, UK, Italy, and Sweden [23]. A study by Zhang et al. [24], evaluated the accuracy of the Gail model and the Tyrer-Cuzick model in predicting breast cancer risk among Chinese women. The study demonstrated that both models exhibited reasonable predictive performance, supporting their applicability in Chinese populations. Given the genetic, epidemiological, and lifestyle similarities between Chinese and Taiwanese populations, these findings suggest that the Gail model may also be a valuable tool for breast cancer risk assessment in Taiwan. However, further empirical validation studies within the Taiwanese population are necessary to confirm its predictive accuracy and clinical utility. Furthermore, recent data indicate an alarming increase in early-onset breast cancers globally, including significant trends observed in Taiwan. This study aims to address these critical gaps by exploring the breast cancer risk score using the Gail model and its relationship to biopsychosocial factors particularly among different age groups in this high-risk population.

## Methods

### Study population

The present study was a cross-sectional study from secondary data of the Taiwan Biobank in 2008 to 2018. The Taiwan Biobank is a government-funded research project that gathers extensive phenotypic measures and genetic data from the Taiwanese population in order to create a prospective cohort study [25]. The recruitment process for Taiwan Biobank involves two components: a community-based arm and a hospital-based collection. The community-based branch has been enrolling individuals between the ages of 30 and 70, who have not been previously diagnosed with cancer, from over 30 recruitment locations around Taiwan [25]. The distribution of these sites is based on the population density of various counties and cities. During the recruitment process, participants were required to give written informed consent and had their first data collected by questionnaires, physical examination, and tests on their blood and urine samples [25].

This study included Taiwanese women who were cancer-free at the time of enrollment from the local population in a community setting that enrolls participants from more than 30 locations in Taiwan. Women between the ages of 35 and 70, both with and without a family history of breast cancer from first-degree relatives, were eligible for enrollment. In total, 149 individuals were excluded from the current investigation because they were under the age of 35.; hence the data for 3,060 healthy women were included in this study.

### Ethical consideration

The investigation was conducted using the Taiwan Biobank data. Within the Taiwan Biobank, the application will undergo rigorous scientific and ethical evaluations conducted by external experts in the corresponding scientific disciplines, as well as the Ethics and Governance Committee (EGC) of Taiwan Biobank [25]. The current study gained ethical approval from the Taipei Medical University Joint Institutional Review Board (No. N201804027). All procedures performed in this study involving human participants were conducted in accordance with Declaration of Helsinki.

### Data collection

In the Taiwan Biobank, phenotypic data primarily relies on self-reporting methods. At recruitment, participants provided written informed consent and had their baseline data collected through questionnaires [25]. A structured questionnaire administered during interviews gathered data on demographic traits, lifestyle choices, environmental exposures, family medical history, and health status [25]. In the current study, we focused on exploring the questionnaire encompassed three main categories related to breast cancer risk factors. Firstly, biological factors such as age [7], BMI, reproductive characteristics (age at menarche, having pregnancy, age at first pregnancy, birth experience, age at first life birth, breastfeeding practice and menopause) [9], and first-degree relatives with breast cancer (mother and/or sister) [8, 26]. Secondly, psychological and behavioral factors like depression status (yes/no) alcohol consumption (never or occasional/ not recently/ recently), smoking habits (yes/no), exposure to secondhand smoke (yes/no), and engagement in physical exercise (yes/no) [10, 11]. Lastly, social factors include marital status (unmarried/married), educational level (primary school/ high school above), and dependency status (living alone/not living alone) [7, 8].

The Gail Model employed to assess risk in our research is a software designed by the National Cancer Institute (NCI) in the United States and has been tested in large populations which provide an accurate estimate of breast cancer risk [22, 27]. This study employed the Breast Cancer Risk Assessment Tool (BCART) created by NCI. The online risk calculator can be accessible through the website https://bcrisktool.cancer.gov/calculator.html. This calculator collects data on various factors including the individual’s age (35–85 years old), age at the onset of menstruation (7–11, 12–13, and ≥ 14 years), age at the first live birth of a child (unknown, no children, < 20, 20–24, 25–29, and ≥ 30 years), number of first-degree relatives (mother, sisters, daughters) with breast cancer, number of previous breast biopsies (whether positive or negative), and presence of atypical hyperplasia in a biopsy (unknown, yes, no) [27]. Females with a Gail Model score exceeding 1.66% were classified as high-risk, while those below this threshold were labeled as low-risk of breast cancer over the next 5 years [22, 28].

### Statistical analysis

We utilized the Statistical Package for the Social Sciences (SPSS), version 27.0 (SPSS Inc., Chicago, IL, USA) to conduct our statistical analysis. Intergroup significance was assessed using Chi-square and Fisher test for categorical. In the current study, a Logistic regression test was performed using the entry approach to evaluate the magnitude of the association between the statistically significant biopsychosocial factors and the level of breast cancer risk. The association was measured using odds ratios (OR) along with a 95% confidence interval (CI). *P*-values below 0.05 are commonly used to indicate statistical significance and were designed as potential risk factors for breast cancer. All analyses were adjusted for potential factors and stratified for age. Furthermore, we utilized the logistic regression model to present the Area Under the Curve (AUC). The curve will provide a clearer understanding of how the biopsychosocial factors enhance predictive accuracy along with relevant statistical details and visual representations of the Receiver Operating Characteristic (ROC) curves. The classification of AUC values were interpreted as excellent (0.9 ≤ AUC), considerable (0.8 ≤ AUC < 0.9), fair (0.7 ≤ AUC < 0.8), poor (0.6 ≤ AUC < 0.7), and fail (0.6 ≤ AUV < 0.5) respectively [29].

## Results

Table 1 presents the distribution of breast cancer risk levels among the participants, as determined by the Gail model. Based on the statistics, 34.2% of the female population in the FHBC group (574 out of 1,676) were found to have a high probability of developing breast cancer, and 495 of these individuals belong to women with FHBC ≥ 50 years old. The determination was made based on a score exceeding 1.66%, which is the standard threshold for identifying high risk in the Gail model. Meanwhile, all of the women in the non-FHBC group (1,384 individuals) were categorized as having a low probability of developing breast cancer. Furthermore, there was a statistically significant difference in breast cancer risk scores between the FHBC and non-FHBC groups (*p* < 0.001).

**Table 1:** The distribution of participants according to the level of the breast cancer risk score based on the Gail model stratified by age (*n* = 3060)

Level of BC Risk	*All women (n = 3060)*			*Women < 50 years old (n = 1237)*			*Women ≥ 50 years old (n = 1823)*		
	FHBC n (%)	Non-FHBC n (%)	*p*-value	FHBC n (%)	Non-FHBC n (%)	*p*-value	FHBC n (%)	Non-FHBC n (%)	*p*-value
Gail model score:									
High-risk (≥ 1.66%)	574 (34.2)	0 (0.0)	**< 0.001**	79 (10.8)	0 (0.0)	**< 0.001**	495 (52.5)	0 (0.0)	**< 0.001**
Low-risk (< 1.66%)	1,102 (65.8)	1,384 (100)		654 (89.2)	504 (100)		448 (47.5)	880 (100)	
**Total**	1,676 (100)	1,384 (100)		733 (100)	504 (100)		943 (100)	880 (100)	

BC: breast cancer; FHBC: family history of breast cancer

The study examined personal information factors to assess the risk of developing invasive breast cancer. The results showed that there were significant associations between age at first live birth, breast cancer in mother and sister, and the presence of FHBC stratified with age. The *p*-values for these associations were < 0.001, < 0.001, and < 0.001, respectively (Table 2). However, two out of the six variables related to personal information (prior breast biopsy and presence of atypical hyperplasia in biopsy) were unknown due to the certainty that all participants were healthy women without breast cancer. Therefore, Taiwan Biobank is unable to offer the data.

**Table 2:** The distribution of participants according to the personal information components of the Gail model, stratified by age (*n* = 3060)

Variables	*All women (n = 3060)*			*Women < 50 years old (n = 1237)*			*Women ≥ 50 years old (n = 1823)*			
	FHBC n (%)	Non-FHBC n (%)	*p*-value	FHBC n (%)	Non-FHBC n (%)	*p*-value	FHBC n (%)		Non-FHBC n (%)	*p*-value
Age at menarche										
7–11	177 (10.6)	156 (11.3)	0.289	87 (11.9)	78 (15.5)	0.055	90 (9.5)		78 (8.9)	0.881
12–13	768 (45.8)	595 (43.0)		377 (51.4)	228 (45.2)		391 (41.5)		367 (41.7)	
≥ 14	731 (43.6)	633 (45.7)		269 (36.7)	198 (39.3)		462 (49.0)		435 (49.4)	
Age at 1st live birth										
No birth	134 (8.0)	215 (15.5)	**< 0.001**	87 (11.9)	114 (22.6)	**< 0.001**	47 (5.0)		101 (11.5)	**< 0.001**
< 20	221 (13.2)	50 (3.6)		112 (15.3)	10 (2.0)		109 (11.6)		40 (4.5)	
20–35	1,273 (76.0)	1,089 (78.7)		502 (68.5)	366 (72.6)		771 (81.8)		723 (82.2)	
> 35	48 (2.8)	30 (2.2)		32 (4.3)	14 (2.8)		16 (1.7)		16 (1.8)	
First-degree relatives:										
Breast cancer mother										
No	846 (50.5)	1,384 (100.0)	**< 0.001**	300 (40.9)	504 (100.0)	**< 0.001**	546 (57.9)		880 (100.0)	**< 0.001**
Yes	830 (49.5)	0 (0.0)		433 (59.1)	0 (0.0)		397 (42.1)		0 (0.0)	
Breast cancer sisters										
No	801 (47.8)	1,384 (100.0)	**< 0.001**	421 (57.4)	504 (100.0)	**< 0.001**	380 (40.3)		880 (100.0)	**< 0.001**
Yes	875 (52.2)	0 (0.0)		312 (42.6)	0 (0.0)		563 (59.7)		0 (0.0)	
Previous breast biopsy										
Yes	0 (0.0)	0 (0.0)	N/A	0 (0.0)	0 (0.0)	N/A	0 (0.0)		0 (0.0)	N/A
No	0 (0.0)	0 (0.0)		0 (0.0)	0 (0.0)		0 (0.0)		0 (0.0)	
Unknown	1,676 (100.0)	1,384 (100.0)		807 (100.0)	540 (100.0)		869 (100.0)		844 (100.0)	
Presence of Atypical hyperplasia in biopsy										
Yes	0 (0.0)	0 (0.0)	N/A	0 (0.0)	0 (0.0)	N/A	0 (0.0)		0 (0.0)	N/A
No	0 (0.0)	0 (0.0)		0 (0.0)	0 (0.0)		0 (0.0)		0 (0.0)	
Unknown	1,676 (54.8)	1,384 (45.2)		807 (100.0)	540 (100.0)		869 (100.0)		844 (100.0)	

**Table 3:** Association between biopsychosocial factors and level of breast cancer risk, stratified by age (*n* = 3060)

Variables	*All women (n = 3060)*			*Women < 50 years old (n = 1237)*			*Women ≥ 50 years old (n = 1823)*			
	Low-risk n (%)	High-risk n (%)	*p*-value	Low-risk n (%)	High-risk n (%)	*p*-value	Low-risk n (%)		High-risk n (%)	*p*-value
*Biological factors*										
BMI										
< 30	2,339 (94.1)	541 (94.3)	0.880	1,088 (94.0)	75 (94.9)	0.722	1,251 (94.2)		466 (94.1)	0.961
≥ 30	147 (5.9)	33 (5.7)		70 (6.0)	4 (5.1)		77 (5.8)		29 (5.9)	
Having pregnancy										
No	335 (13.5)	54 (9.4)	**0.008**	228 (19.7)	4 (5.1)	**0.001**	107 (8.1)		50 (10.1)	0.167
Yes	2,151 (86.5)	520 (90.6)		930 (80.3)	75 (94.9)		1,221 (91.9)		445 (89.9)	
Age at 1st pregnancy										
No pregnant	335 (13.5)	54 (9.4)	**< 0.001**	228 (19.7)	4 (5.1)	**< 0.001**	107 (8.1)		50 (10.1)	**< 0.001**
< 20	327 (13.1)	20 (3.5)		138 (11.9)	4 (5.1)		189 (14.2)		16 (3.3)	
20–35	1,787 (71.9)	485 (84.5)		767 (66.2)	67 (84.8)		1,020 (76.8)		418 (84.4)	
> 35	37 (1.5)	15 (2.6)		25 (2.2)	4 (5.1)		12 (0.9)		11 (2.2)	
Birth experience										
No	408 (16.4)	67 (11.7)	**0.005**	278(24.0)	4 (5.1)	**< 0.001**	130 (9.8)		63 (12.7)	**0.070**
Yes	2,078 (83.6)	507 (88.3)		880 (76.0)	75 (94.9)		1,198 (90.2)		432 (87.3)	
Breastfeeding practice										
Never	194 (7.8)	66 (11.5)	**0.014**	164 (14.2)	4 (5.1)	**0.009**	30 (2.3)		62 (12.5)	**< 0.001**
No	886 (35.6)	203 (35.4)		322 (27.8)	16 (20.3)		564 (42.5)		187 (37.8)	
Yes	1,406 (56.6)	305 (53.1)		672 (58.0)	59 (74.7)		734 (55.2)		246 (49.7)	
Menopause										
No	1,343 (54.0)	172 (30.0)	**< 0.001**	1,072 (92.6)	71 (89.9)	0.381	271 (20.4)		101 (20.4)	0.999
Yes	1,143 (46.0)	402 (70.0)		86 (7.4)	8 (10.1)		1,057 (79.6)		394 (79.6)	
*Psychological and behavioral factors*										
Depression status										
No	2,356 (94.8)	548(95.5)	0.492	1,106 (95.5)	76 (96.2)	0.772	1,250 (94.1)		472 (95.4)	0.308
Yes	130 (5.2)	26 (4.5)		52 (4.5)	3 (3.8)		78 (5.9)		23 (4.6)	
Alcohol consumption										
Never / occasionally	2,427 (97.6)	561 (97.7)	0.091	1,129 (97.5)	77 (97.5)	0.772	1,298 (97.7)		484 (97.8)	**0.065**
Not recently	17 (0.7)	0 (0.0)		6 (0.5)	0 (0.0)		11 (0.8)		0 (0.0)	
Recently	42 (1.7)	13 (2.3)		23 (2.0)	2 (2.5)		19 (1.5)		11 (2.2)	
Smoking										
No	2,244 (90.3)	516 (89.9)	0.788	1,016 (87.7)	58 (86.1)	0.664	1,128 (92.5)		448 (90.5)	0.171
Yes	242 (9.7)	58 (10.1)		142 (12.3)	11 (13.9)		100 (7.5)		47 (9.5)	
Secondhand smoke										
No	2,290 (92.1)	553 (96.3)	**< 0.001**	1,048 (90.5)	77 (97.5)	**0.037**	1,242 (93.5)		476 (96.2)	**0.032**
Yes	196 (7.9)	21 (3.7)		110 (9.5)	2 (2.5)		86 (6.5)		19 (3.8)	
Exercise										
No	1,483 (59.7)	301 (52.4)	**0.002**	851 (73.5)	60 (75.9)	0.631	632 (47.6)		241 (48.7)	0.677
Yes	1,003 (40.3)	273 (47.6)		307 (26.5)	19 (24.1)		696 (52.4)		254 (51.3)	
*Social factors*										
Marital Status										
Unmarried	300 (12.1)	44 (7.7)	**0.003**	220 (19.0)	5 (6.3)	**0.005**	80 (6.0)		39 (7.9)	0.154
Married	2,186 (87.9)	530 (92.3)		938 (81.0)	74 (93.7)		1,248 (94.0)		456 (92.1)	
Educational level										
Primary school	294 (11.8)	43 (7.5)	**0.003**	45 (3.9)	0 (0.0)	0.074	249 (18.8)		43 (8.7)	**< 0.001**
High school or above	2,191 (88.2)	531 (92.5)		1,113 (96.1)	79 (100.0)		1,079 (81.3)		452 (91.3)	
Job experience										
No	1,720 (69.2)	440 (76.7)	**< 0.001**	813 (70.2)	56 (70.9)	0.898	907 (68.3)		384 (77.6)	**< 0.001**
Yes	766 (30.8)	134 (23.3)		345 (29.8)	23 (29.1)		421 (31.7)		111 (22.4)	
Dependency										
Not live alone	2,250 (90.5)	528 (92.0)	0.269	1,083 (93.5)	77 (6.5)	0.160	1,167 (87.9)		451 (91.1)	0.052
Live alone	236 (9.5)	46 (8.0)		75 (6.5)	2 (2.5)		161 (12.1)		44 (8.9)	

**Table 4:** Evaluation the risk of breast cancer according to the biopsychosocial factors stratified by age

Variables	Outcome: High-Risk			
	Women < 50 years old		Women ≥ 50 years old	
	OR (95% CI)	*p*-value	OR (95% CI)	*p*-value
*Biological factors*				
Having pregnancy				
Yes	Ref	**0.001**	Ref	0.122
No	0.19 (0.07 to 0.53)		1.33 (0.93 to 1.89)	
Age at 1st pregnancy				
< 35	Ref	0.055	Ref	**0.021**
≥ 35	3.11 (0.97 to 9.89)		2.69 (1.16 to 6.27)	
Birth experience				
Yes	Ref	**< 0.001**	Ref	**0.044**
No	0.15 (0.06 to 0.41)		1.39 (1.01 to 1.93)	
Breastfeeding practice				
Yes	Ref		Ref	
No	0.70 (0.42 to 1.18)	0.182	1.04 (0.83 to 1.30)	0.729
Never	0.62 (0.17 to 2.18)	0.452	7.65 (4.67 to 12.54)	**< 0.001**
Menopause				
No	Ref	**0.040**	Ref	
Yes	1.89 (1.03 to 3.49)		0.91 (0.69 to 1.19)	0.504
*Psychological and behavioral factors*				
Secondhand smoke				
No	Ref	**0.047**	Ref	0.052
Yes	0.23 (1.16 to 6.27)		0.59 (0.34 to 1.00)	
Exercise				
Yes	Ref	0.599	Ref	0.904
No	1.15 (0.68 to 1.95)		1.01 (0.82 to 1.26)	
*Social factors*				
Marital Status				
Married	Ref	0.820	Ref	0.135
Unmarried	0.78 (0.09 to 6.37)		1.39 (0.90 to 2.14)	
Educational level				
Primary school	Ref	0.122	Ref	**< 0.001**
High school or above	4.79 (0.66 to 34.6)		2.43 (1.72 to 3.43)	
Job experience				
Yes	Ref	0.540	Ref	**< 0.001**
No	1.18 (0.70 to 1.97)		1.58 (1.23 to 2.03)	

The biopsychosocial characteristics of the study population are presented individually in Table 3. Among the six biological factors analyzed, five demonstrated significant variations in breast cancer risk among women in both groups: pregnancy status (*p* = 0.008), age at first pregnancy (*p* < 0.001), birth experience (*p* = 0.005), breastfeeding practices (*p* = 0.014), and menopausal status (*p* < 0.001). Additionally, psychological and behavioral factors, including exposure to secondhand smoke (*p* < 0.001), and exercise habits (*p* = 0.002) exhibited significant differences across all groups rather than within individual groups. Social factors, such as marital status (*p* = 0.003), educational level (*p* = 0.003), and job experience (*p* < 0.001), showed significant differences across all group.

Logistic regression analysis was conducted to examine the substantial predictor of biopsychosocial factors that showed a *p*-value of less than 0.05 on the bivariate analysis. 10 out of 15 variables were selected and taken into consideration in the logistic regression model. Based on Table 4 indicates that several factors serve as potential predictors of breast cancer risk in the older group, including age at first pregnancy (OR: 2.69; 95% CI: 1.16–6.27; *p* = 0.021), birth experience (OR: 1.39; 95% CI: 1.01–1.93; *p* = 0.044), breastfeeding practice (OR: 7.65; 95% CI: 4.67–12.54; *p* < 0.001), educational level (OR: 2.43; 95% CI: 1.72 to 3.43; *p* < 0.001), and job experience (OR: 1.58; 95% CI: 1.23–2.03; *p* < 0.001).

At the same time, significant predictors of breast cancer risk in the younger group include pregnancy status (OR: 0.19; 95% CI: 0.07–0.53; *p* = 0.001), birth experience (OR: 0.15; 95% CI: 0.06–0.41; *p* < 0.001), menopausal status (OR: 1.89; 95% CI: 1.03–3.49; *p* = 0.040), and exposure to secondhand smoke (OR: 0.23; 95% CI: 1.16–6.27; *p* = 0.047). However, there appears to be an inconsistency in the odds ratio (OR) values for significant factors in younger women, particularly pregnancy status, birth experience, and exposure to secondhand smoke, which seem to function as protective factors rather than predictive risk factors.

**Fig. 1:**
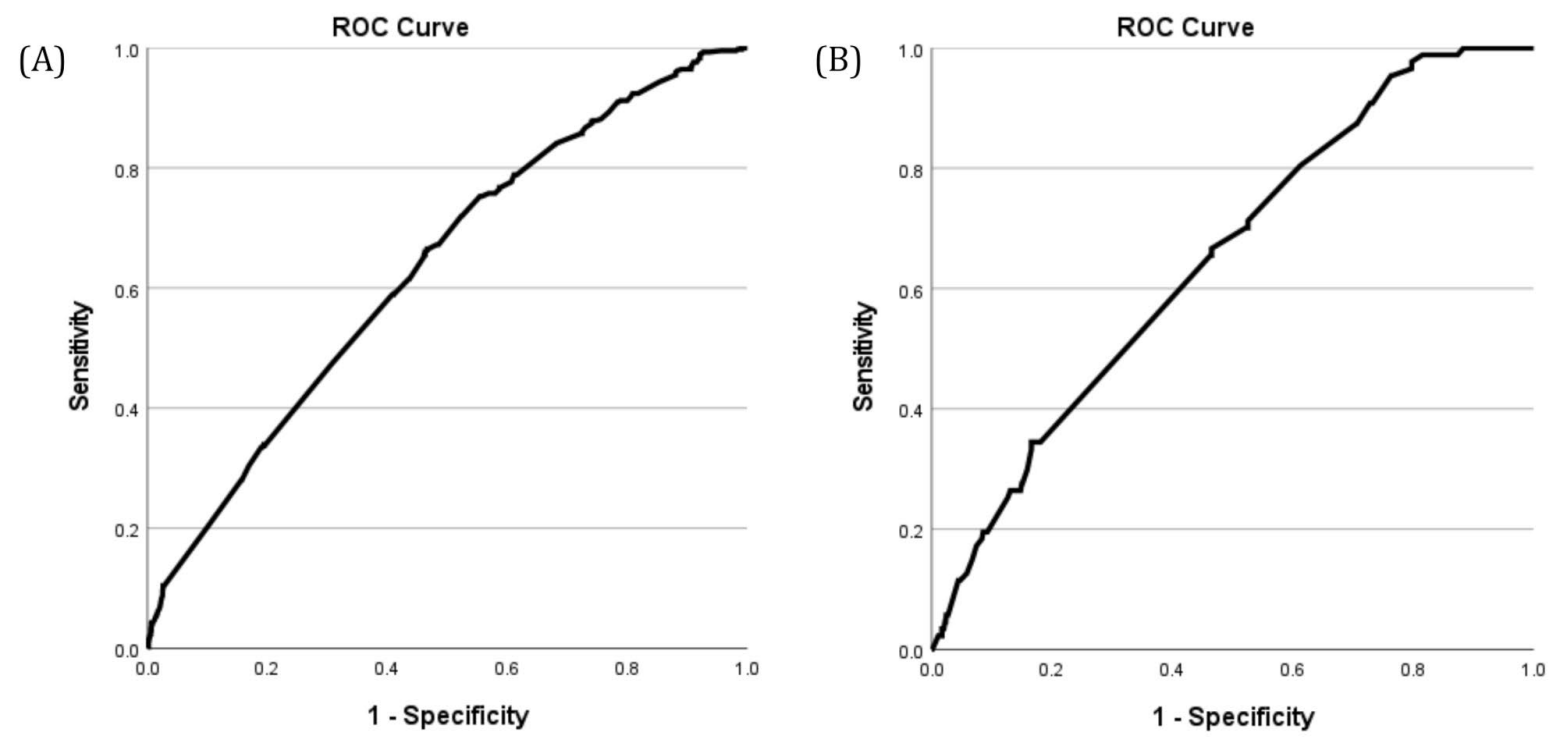
Receiver operating characteristic (ROC) curve analysis for the testing data. (**A**) ROC curve for women aged ≥ 50 years, with an AUC score of 0.633. (**B**) ROC curve for women aged < 50 years, with an AUC score of 0.648. *AUC: Area Under the Curve*

Our study conducted sensitivity and specificity analyses using logistic regression, represented by the Receiver Operating Characteristic (ROC) curve, to assess the accuracy of biopsychosocial factors in predicting breast cancer risk across different age groups. As shown in Fig. 1, the biopsychosocial factors demonstrated poor discriminatory ability in predicting breast cancer risk in both age groups, with AUC scores of 0.633 and 0.648, respectively.

## Discussion

Breast cancer in Taiwan is the most common form of cancer in Taiwanese women and the most frequent cause of cancer-related death [5]. The result of the current study indicates that 34.2% of Taiwanese women with a family history of breast cancer have a high risk of developing breast cancer over the next five years. Moreover, the most important personal information and biopsychosocial factors associated with breast cancer risk, particularly in older women as a high-risk population include age at first live birth, history of breast cancer mother, breast cancer sister, birth experience, breastfeeding practice, educational level, and job experience. Therefore, it’s crucial for both patients and healthcare providers to accurately evaluate an individual’s risk of breast cancer in order to decide on the potential use of prevention strategies for high-risk women. Understanding that women at elevated risk of breast cancer require significant assistance in healthcare decision-making and embracing the impact of various prevention strategies is essential.

Different methods have been created to assess risk, with the Gail model emerging as a commonly utilized tool. This research aimed to assess the reliability of the Gail model in predicting breast cancer development among Taiwanese individuals with a family history of the disease, incorporating individual and additional risk factors. This approach enables prompt risk evaluation for breast cancer during patient examinations, facilitating timely follow-up and treatment planning, contingent upon meeting specific criteria. The primary focus lies in identifying individuals at heightened risk of breast cancer [27].

Breast cancer is a prevalent concern among women aged 45 and older. According to research conducted by Rojas et al. [19] on breast cancer epidemiology and risk factors, the majority of female breast cancer cases are diagnosed in the age range of 55 to 64, with a median diagnosis age of 61 years. Less than 5% of breast cancer diagnoses occur in women under 40 years old, and similar to many other types of cancer, the risk rises with increasing age [30]. In addition, our study established a family history of breast cancer as a significant risk factor for breast cancer. This discovery aligns with previous research, which found that having one first-degree relative with breast cancer is linked to a 1.8-fold increase in risk while having two first-degree relatives with breast cancer is associated with a 2.9-fold higher risk [31, 32].

Aging is accompanied by a considerable reduction of several mechanisms in women’s bodies including estrogen hypersensitivity and mammary epithelial cell changes [33], . Elderly women exhibit markedly decreased levels of circulating estrogens, despite a greatly elevated risk of hormone-dependent cancer. The seeming a contradiction can be elucidated by profound cellular and molecular transformations that take place in the mammary gland following menopause [33]. The process of aging also results in alterations to the breast tissue, specifically the atrophy of ductal-lobular tissue, which occurs at the expense of the connective and adipose stroma. Russo et al. [34] discovered that following menopause, differentiated lobules experiences regress and become undifferentiated lobules similar to those observed before puberty. Additionally, it induces changes in the system responsible for repairing double-stranded DNA by suppressing the ATM protein (ataxia telangiectasia mutated) pathway, potentially enhancing its ability to transform cells [35].

Considering reproductive characteristics, we discovered that, age at first live birth, birth experience, and breastfeeding practice, as significant predictors associated with breast cancer development. Research indicates that giving birth at a later age, specifically > 30 years old, is associated with an increased risk of breast cancer [36, 37]. Albreksten et al. [37] conducted a study revealing that the risk of breast cancer rises with advancing age at first childbirth, beyond what could be attributed to the delayed protective effects of pregnancy, such as the decreasing risk over time since giving birth. Additionally, they observed that the temporary surge in risk shortly after childbirth was most pronounced following a late first childbirth [37]. A prior study shown that a prolonged period between the onset of menstruation and the first occurrence of a live birth also raises the risk of developing breast cancer [38]. The breast tissue is mostly undifferentiated and is sensitive to the mitogenic actions of estrogen receptor (ER) and progesterone receptor (PR) at this time. As a result, it is particularly vulnerable to carcinogens [38, 39]. The period from the onset of menstruation until the first life of birth has been referred to as a “period of high vulnerability” [40]. A reduced time period between the onset of menstruation and the first life of giving birth is associated with elevated quantities of hormones produced within the body [41].

Prior research has demonstrated that mothers who do not breastfeed are at a high risk of reproductive cancers. Specifically, breast, ovarian, and uterine cancers are more prevalent among women who opt not to breastfeed [42]. Conversely, breastfeeding has been linked to a decreased risk of breast cancer. This protective effect appears to be more pronounced among women who breastfeed for extended durations throughout their lives [43]. For instance, women who breastfed for a minimum of 24 months over their lifetimes exhibited a significantly lower risk of developing breast cancer compared to those who breastfed for fewer than 24 months. When compared to women who breastfed for a total of 0–11 months during their lifetimes, there was a 66.3% reduction in breast cancer risk among those who breastfed for 12–23 months, an 87.4% reduction among those who breastfed for 24–35 months, and a 94% reduction among those who breastfed for 36–47 months [42]. A study conducted in the Taiwanese population showed that breastfeeding for more than 3 years displayed significant protective effects against breast cancer [44].

The periodic impact of estrogen and progesterone on breast tissue can be delayed by prolonged breastfeeding or an increased number of pregnancies [45]. Prolonged breastfeeding decreases the likelihood of developing triple-negative malignancies [46]. The term can be described as a strong correlation between slow-paced weaning and mitigation of inflammatory response throughout the involution. Two distinct types of lobules are observed in the breast tissue of mothers undergoing progressive weaning, indicating a gradual replacement of the terminal duct lobular units (TDLUs). One type of cell was actively producing milk, while the other was involution, as indicated by infiltration of CD45-positive acute reactive immune cells and CD68-positive macrophages [45, 47].

Our research demonstrated a correlation between menopause and an increased chance of developing breast cancer in the younger age group. A study conducted by Chow et al. (1997) in Taiwan examined the demographic features and medical aspects of menopausal women. The study found that there were 14,298 newly reported instances of malignant neoplasms in women. Around 60% of these incidents were observed in women who were 50 years of age or older. The median age at which cervix uteri, breast, and ovarian cancers typically arise is approximately 48 to 49 years, which is in close proximity to the age at which menopause usually begins [48]. Throughout women’s reproductive years, which broadly span from the onset of menstruation to menopause, the ovary generates steroid hormones that directly influence the growth and operation of the breast. It is recognized that experiencing late menopause elevates the risk of breast cancer in women [49].

Educational level and job experience are key social determinants that can influence breast cancer risk through multiple pathways. Our study found that higher education was significantly associated with increased breast cancer risk, which may seem counterintuitive at first. However, education itself is not a direct biological predictor of breast cancer; rather, it is a proxy for various lifestyle, reproductive, and healthcare-seeking behaviors that contribute to disease development.

Higher educational attainment is often linked to lifestyle factors such as increased alcohol consumption, delayed childbearing, lower parity (fewer children), and greater use of hormone replacement therapy (HRT)—all of which have been identified as potential risk factors for breast cancer. Previous research suggests that women with higher education levels may have greater health awareness and access to healthcare, leading to earlier and more frequent breast cancer screenings, which could contribute to the observed higher incidence rates in this group [50]. In contrast, lower educational attainment and socioeconomic disadvantage have been associated with poorer breast cancer outcomes rather than lower risk. Vona-Davis and Rose [51] highlighted that social deprivation is linked to a higher incidence of aggressive breast cancer subtypes, such as estrogen receptor (ER)-negative and triple-negative breast cancer, which are often diagnosed at later stages with worse prognoses. Additionally, socioeconomic disparities—characterized by low family income, limited education, and lack of health insurance—can lead to reduced participation in mammography screening programs and delays in seeking medical care, ultimately contributing to poorer survival rates [51, 52].

Employment status is another crucial factor influencing breast cancer risk and outcomes. Stable employment is often associated with better healthcare access, including employer-sponsored insurance, routine medical check-ups, and preventive screenings. Conversely, job insecurity or unemployment may increase stress levels, limit healthcare access, and reduce the likelihood of engaging in health-promoting behaviors, all of which can negatively impact breast cancer detection and prognosis [53]. Therefore, our findings highlight the complex relationship between education, employment, and breast cancer risk, emphasizing the need for public health strategies that address both ends of the socioeconomic spectrum. While higher education is associated with lifestyle-related risk factors, lower education and economic disadvantage contribute to delayed diagnosis and worse outcomes, necessitating targeted interventions to improve both prevention and early detection efforts across diverse populations.

### Strength and limitation

This study utilized a substantial sample obtained from the Taiwan Biobank, which adequately captured the Taiwanese population. In addition, the Gail Model has been tested in large populations worldwide and has been shown to provide accurate estimates of breast cancer risk. Moreover, this study focuses on investigating women who have a family history of breast cancer in their first relative as a vulnerable group for developing breast cancer stratified by age. Therefore, the study results could be applied as recommendations for the prevention programs targeting this particular population.

However, there are numerous limitations to consider when adopting these investigations. First, the study method was cross-sectional because the variables included in the study was obtained only from the health questionnaire of the of Taiwan Biobank at a single point in time. Consequently, making it difficult to establish cause-and-effect relationships. Therefore, in future studies, the inclusion of an appropriate dataset would enable a longitudinal study method to validate breast cancer-related factors among women who have been diagnosed with breast cancer. Second, all of individuals in this study are healthy women who do not have the personal information related a history of biopsy and presence of atypical hyperplasia in biopsy relevant to the data. It may impact the accuracy of our findings. Future studies should aim to collect comprehensive personal data, including biopsy history and the presence of atypical hyperplasia, to enhance the accuracy and applicability of breast cancer risk assessment models.

Third, an inconsistency was observed in the odds ratio (OR) values for significant factors in younger women, particularly pregnancy status, birth experience, and exposure to secondhand smoke, which appear to act as protective factors rather than predictive risk factors. Variations in physiological responses to reproductive and environmental exposures across age groups may contribute to these findings. Future research with larger, more diverse cohorts and comprehensive adjustments for confounding factors is necessary to validate these results and clarify the underlying mechanisms. Fourth, the poor discriminatory ability of the biopsychosocial factors in predicting breast cancer risk, as reflected in the relatively low AUC scores both age groups. This suggests that while these factors may contribute to breast cancer risk, they are insufficient as standalone predictors. The limited predictive performance may be due to the complex and multifactorial nature of breast cancer and may be influenced by biases stemming from the use of self-reported data. It can result in certain weaknesses, such as a lack of objectivity and the inaccuracy of data. Lastly, although previous studies have assessed the predictive performance of the Gail model in Chinese populations, the absence of validation studies conducted exclusively in Taiwan raises concerns regarding potential differences in genetic, environmental, and lifestyle factors that could influence model accuracy. Large-scale, population-based cohort studies should be carried out to evaluate how well the model performs in predicting breast cancer risk among Taiwanese women, considering potential variations in genetic predisposition, reproductive factors, lifestyle influences, and environmental exposures unique to Taiwan. Ultimately, such research would provide critical insights to refine risk prediction tools and optimize breast cancer screening and prevention strategies tailored to the Taiwanese demographic.

## Conclusion

The present research indicates that the Gail model assesses individual breast cancer risk among Taiwanese women. 34.2% of women with a family history of breast cancer have a high-risk score of developing breast cancer over the next five years. Various biopsychosocial factors serve as predictors of breast cancer development, particularly in the older group, including age at first pregnancy, birth experience, breastfeeding practice, educational level, and job experience. These findings suggest that targeted interventions (i.e. secondary prevention) such as screening, mammography, counseling, educational interventions, and breast self-examination based on high-risk level and biopsychosocial factors could be implemented to prevent breast cancer, particularly among Taiwanese individuals with a family history of the disease.

## Data Availability

The datasets generated and/or analyzed during the current study are not publicly available due to privacy and ethical restrictions imposed by the Taiwan Biobank, but are available from the corresponding author on reasonable request and with permission from the Taiwan Biobank.

## Abbreviations

AUC: Area Under Curve
BC: Breast cancer
BCRAT: Breast Cancer Risk Assessment Tool
BMI: Body mass index
CI: Confidence interval
FHBC: Family history of breast cancer
OR: Odds ratio
ROC: Receiver Operating Characteristic

## References

[1] Sung H, et al. Global cancer statistics 2020: GLOBOCAN estimates of incidence and mortality worldwide for 36 cancers in 185 countries. CA Cancer J Clin. 2021;71(3):209–49. 33538338 10.3322/caac.21660

[2] Bray F, et al. Global cancer statistics 2022: GLOBOCAN estimates of incidence and mortality worldwide for 36 cancers in 185 countries. CA Cancer J Clin. 2024;74(3):229–63. 38572751 10.3322/caac.21834

[3] Center TCR. In: Administration HP, editor. Incidence and mortality rates for the top 10 cancer in Taiwan, 2021. Editor: Taiwan; 2021.

[4] He J, Chen W. Chinese cancer registry annual report. Military Medical Science Press, Beijing, 2012:12:56–58.

[5] Hou IC, et al. Quality of life of women after a first diagnosis of breast cancer using a self-management support mHealth app in Taiwan: randomized controlled trial. JMIR Mhealth Uhealth. 2020;8(3):e17084. 32130181 10.2196/17084 PMC7081131

[6] Thakur C, et al. Epigenetics and environment in breast cancer: new paradigms for anti-cancer therapies. Front Oncol. 2022;12:971288. 36185256 10.3389/fonc.2022.971288 PMC9520778

[7] Holm J, et al. Assessment of breast cancer risk factors reveals subtype heterogeneity. Cancer Res. 2017;77(13):3708–17. 28512241 10.1158/0008-5472.CAN-16-2574

[8] Sun YS, et al. Risk factors and preventions of breast cancer. Int J Biol Sci. 2017;13(11):1387–97. 29209143 10.7150/ijbs.21635 PMC5715522

[9] Engmann NJ, et al. Population-attributable risk proportion of clinical risk factors for breast cancer. JAMA Oncol. 2017;3(9):1228–36. 28152151 10.1001/jamaoncol.2016.6326 PMC5540816

[10] Hoxha I, et al. Breast cancer and lifestyle factors: umbrella review. Hematol Oncol Clin North Am. 2024;38(1):137–70. 37635047 10.1016/j.hoc.2023.07.005

[11] Lester SP, Kaur AS, Vegunta S. Association between lifestyle changes, mammographic breast density, and breast cancer. Oncologist. 2022;27(7):548–54. 35536728 10.1093/oncolo/oyac084 PMC9256023

[12] McPherson K, Steel CM, Dixon JM. ABC of breast diseases. Breast cancer–epidemiology, risk factors and genetics. BMJ. 1994;309(6960):1003–6. 7950693 10.1136/bmj.309.6960.1003 PMC2541301

[13] Radecka B, Litwiniuk M. Breast cancer in young women. Ginekol Pol. 2016;87(9):659–63. 27723074 10.5603/GP.2016.0062

[14] Brewer HR, et al. Family history and risk of breast cancer: an analysis accounting for family structure. Breast Cancer Res Treat. 2017;165(1):193–200. 28578505 10.1007/s10549-017-4325-2 PMC5511313

[15] Liu L, et al. Correlation between family history and characteristics of breast cancer. Sci Rep. 2021;11(1):6360. 33737705 10.1038/s41598-021-85899-8 PMC7973811

[16] Winters S, et al. Breast cancer epidemiology, prevention, and screening. Prog Mol Biol Transl Sci. 2017;151:1–32. 29096890 10.1016/bs.pmbts.2017.07.002

[17] Al Otaibi HH. Breast cancer risk assessment using the Gail model and it’s predictors in Saudi women. Asian Pac J Cancer Prev. 2017;18(11):2971–5. 29172267 10.22034/APJCP.2017.18.11.2971 PMC5773779

[18] Gail MH, et al. Projecting individualized probabilities of developing breast cancer for white females who are being examined annually. J Natl Cancer Inst. 1989;81(24):1879–86. 2593165 10.1093/jnci/81.24.1879

[19] Costantino JP, et al. Validation studies for models projecting the risk of invasive and total breast cancer incidence. J Natl Cancer Inst. 1999;91(18):1541–8. 10491430 10.1093/jnci/91.18.1541

[20] Del Valle Peña, Colmenares J, et al. Is using the Gail model to calculate the risk of breast cancer in the Venezuelan population justified? Ecancermedicalscience. 2023;17:1590. 37799948 10.3332/ecancer.2023.1590 PMC10550297

[21] Adebayo R, et al. Assessment of breast cancer risk factors among women of reproductive age group in Oshogbo using Gail model. Int J Nurs Midwifery. 2019;11:7–17.

[22] Abdulhussain Fadhil A, et al. Identify breast cancer risk factors using the Gail assessment model in Iraq. Arch Razi Inst. 2022;77(5):1901–7. 37123119 10.22092/ARI.2022.359509.2436 PMC10133634

[23] Clendenen TV, et al. Breast cancer risk prediction in women aged 35–50 years: impact of including sex hormone concentrations in the Gail model. Breast Cancer Res. 2019;21(1):42. 30890167 10.1186/s13058-019-1126-z PMC6425605

[24] Zhang L, et al. Use of receiver operating characteristic (ROC) curve analysis for Tyrer-Cuzick and Gail in breast Cancer screening in Jiangxi Province, China. Med Sci Monit. 2018;24:5528–32. 30089770 10.12659/MSM.910108 PMC6097135

[25] Feng YA, et al. Taiwan biobank: a rich biomedical research database of the Taiwanese population. Cell Genom. 2022;2(11):100197. 36776991 10.1016/j.xgen.2022.100197 PMC9903657

[26] Majeed W, et al. Breast cancer: major risk factors and recent developments in treatment. Asian Pac J Cancer Prev. 2014;15(8):3353–8. 24870721 10.7314/apjcp.2014.15.8.3353

[27] Gail MH, Greene MH. Gail model and breast cancer. Lancet. 2000;355(9208):1017. 10768462 10.1016/S0140-6736(05)74761-9

[28] Saleh B, et al. Gail model utilization in predicting breast cancer risk in Egyptian women: a cross-sectional study. Breast Cancer Res Treat. 2021;188(3):749–58. 33852122 10.1007/s10549-021-06200-z

[29] Çorbacıoğlu ŞK, Aksel G. Receiver operating characteristic curve analysis in diagnostic accuracy studies: a guide to interpreting the area under the curve value. Turkish J Emerg Med. 2023;23(4). 10.4103/tjem.tjem_182_23 PMC10664195 38024184

[30] Rojas K, Stuckey A. Breast cancer epidemiology and risk factors. Clin Obstet Gynecol. 2016;59(4):651–72. 27681694 10.1097/GRF.0000000000000239

[31] Collaborative Group on Hormonal Factors in Breast Cancer. Familial breast cancer: collaborative reanalysis of individual data from 52 epidemiological studies including 58 209 women with breast cancer and 101 986 women without the disease. Lancet. 2001;358(9291):1389–99. https://pubmed.ncbi.nlm.nih.gov/11705483/. 10.1016/S0140-6736(01)06524-2 11705483

[32] Zhang Y, et al. Analysis of breast cancer family history, Estrogen receptor status, and breast Cancer outcomes in Sweden. JAMA Netw Open. 2023;6(6):e2318053. 37310740 10.1001/jamanetworkopen.2023.18053 PMC10265300

[33] Lodi M, et al. Breast cancer in elderly women and altered clinico-pathological characteristics: a systematic review. Breast Cancer Res Treat. 2017;166(3):657–68. 28803352 10.1007/s10549-017-4448-5

[34] Russo J, Russo IH. Development of the human breast. Maturitas. 2004;49(1):2–15. 15351091 10.1016/j.maturitas.2004.04.011

[35] Raynaud CM, et al. DNA damage repair and telomere length in normal breast, preneoplastic lesions, and invasive cancer. Am J Clin Oncol. 2010;33(4):341–5. 19884805 10.1097/COC.0b013e3181b0c4c2

[36] Aurin J, Thorlacius H, Butt ST. Age at first childbirth and breast cancer survival: a prospective cohort study. BMC Res Notes. 2020;13(1):9. 31907014 10.1186/s13104-019-4864-1 PMC6945722

[37] Albrektsen G, et al. Breast cancer risk by age at birth, time since birth and time intervals between births: exploring interaction effects. Br J Cancer. 2005;92(1):167–75. 15597097 10.1038/sj.bjc.6602302 PMC2361726

[38] Huang Z, et al. Associations of reproductive time events and intervals with breast cancer risk: a report from the Shanghai breast cancer study. Int J Cancer. 2014;135(1):186–95. 24323821 10.1002/ijc.28644 PMC4591050

[39] Russo J, Russo IH. Role of differentiation in the pathogenesis and prevention of breast cancer. Endocrine-related Cancer. 1997;4(1):7–21.

[40] McDougall JA, et al. Timing of menarche and first birth in relation to risk of breast cancer in A-bomb survivors. Cancer Epidemiol Biomarkers Prev. 2010;19(7):1746–54. 20570914 10.1158/1055-9965.EPI-10-0246

[41] Iversen A, et al. Ovarian hormones and reproductive risk factors for breast cancer in premenopausal women: the Norwegian EBBA-I study. Hum Reprod. 2011;26(6):1519–29. 21467202 10.1093/humrep/der081 PMC3096559

[42] do Carmo França-Botelho A, et al. Breastfeeding and its relationship with reduction of breast cancer: a review. Asian Pac J Cancer Prev. 2012;13(11):5327–32. 23317179

[43] Freund C, et al. Breastfeeding and breast cancer. Gynecol Obstet Fertil. 2005;33(10):739–44. 16139543 10.1016/j.gyobfe.2005.07.030

[44] Lai FM, et al. A case-control study of parity, age at first full-term pregnancy, breast feeding and breast cancer in Taiwanese women. Proc Natl Sci Counc Repub China B. 1996;20(3):71–7. 8956522

[45] Kobayashi S, et al. Reproductive history and breast cancer risk. Breast Cancer. 2012;19(4):302–8. 22711317 10.1007/s12282-012-0384-8 PMC3479376

[46] Gaudet MM, et al. Risk factors by molecular subtypes of breast cancer across a population-based study of women 56 years or younger. Breast Cancer Res Treat. 2011;130(2):587–97. 21667121 10.1007/s10549-011-1616-x PMC3721192

[47] O’Brien J, et al. Alternatively activated macrophages and collagen remodeling characterize the postpartum involuting mammary gland across species. Am J Pathol. 2010;176(3):1241–55. 20110414 10.2353/ajpath.2010.090735 PMC2832146

[48] Chow SN, Huang CC, Lee YT. Demographic characteristics and medical aspects of menopausal women in Taiwan. J Formos Med Assoc. 1997;96(10):806–11. 9343980

[49] Collaborative Group on Hormonal Factors in Breast Cancer. Menarche, menopause, and breast cancer risk: individual participant meta-analysis, including 118 964 women with breast cancer from 117 epidemiological studies. Lancet Oncol. 2012;13(11):1141–51. https://pubmed.ncbi.nlm.nih.gov/23084519/. 10.1016/S1470-2045(12)70425-4 PMC3488186 23084519

[50] Dong J-Y, Qin L-Q. Education level and breast cancer incidence: a meta-analysis of cohort studies. Menopause. 2020;27(1). https://pubmed.ncbi.nlm.nih.gov/31479033/. 10.1097/GME.0000000000001425 31479033

[51] Vona-Davis L, Rose DP. The influence of socioeconomic disparities on breast cancer tumor biology and prognosis: a review. J Womens Health (Larchmt). 2009;18(6):883–93. 19514831 10.1089/jwh.2008.1127

[52] Dailey AB, et al. Neighborhood-level socioeconomic predictors of nonadherence to mammography screening guidelines. Cancer Epidemiol Biomarkers Prev. 2007;16(11):2293–303. 18006918 10.1158/1055-9965.EPI-06-1076

[53] Hamlish T, et al. Impact of a breast cancer diagnosis on finances and marital status in young women. BMC Womens Health. 2025;25(1):86. 39994652 10.1186/s12905-025-03607-4 PMC11853482

